# Attachment styles and healthcare utilization: exploring the role of the patient-doctor relationship

**DOI:** 10.1186/s12913-023-10484-w

**Published:** 2024-01-11

**Authors:** I. Schmalbach, G. H. Franke, W. Häuser, B. Strauss, K. Petrowski, Elmar Brähler

**Affiliations:** 1 Department of Medical Psychology and Medical Sociology, Johannes-Gutenberg University Mainz, Mainz, Germany; 2 Psychology of Rehabilitation, University of Applied Sciences Magdeburg and Stendal, Magdeburg, Germany; 3 Innere Medizin I, Klinikum Saarbrücken gGmbH, Winterberg 1, 66119 Saarbrücken, Germany; 4 Institut für Psychosoziale Medizin und Psychotherapie, Klinikum der Friedrich-Schiller-Universität, Universitätsklinikum Jena, Jena, Germany; 5 Faculty of Medicine Carl Gustav Carus, Department of General Practice/MK3, Technische Universität Dresden, Dresden, Germany; 6 Integrated Research and Treatment Center (IFB) Adiposity Diseases – Behavioral Medicine, Medical Psychology and Medical Sociology, University of Leipzig Medical Center, Leipzig, Germany; 7 University Medical Centre, Johannes Gutenberg University Mainz, Mainz, Germany

**Keywords:** Attachment-style, Healthcare utilization, Patient-doctor-relationship, Behavior patterns, Medical care

## Abstract

**Background:**

With the purpose of improving healthcare, past research has examined the link between healthcare utilization and attachment. It is suggested that an individual’s attachment style influences both the quality of their patient-physician relationship and healthcare utilization patterns. Nevertheless, most studies concentrate on the individual aspect, overlooking the dyadic dimension; specifically, the investigation of how insecure attachment relates to health behavior within patient-physician relationships. This gap leaves the role of the patient-doctor relationship in this process unclear. Therefore, to elucidate this complex interplay, we hypothesized that the correlation between attachment and healthcare utilization is mediated by the quality of the patient-physician-relationship.

**Method:**

Participant selection was based on electoral districts, a random-route procedure, and the Kish selection grid. The participants were visited by a trained interviewer who collected psychometric and sociodemographic information. Participants answered the Experiences in Close Relationships-Revised questionnaire (ECR-RD8) and the Patient-Doctor Relationship Questionnaire (PDRQ-9). Additionally, participants were asked about their healthcare utilization. The final sample consisted of *N* = 2.275 participants.

**Results:**

In average the participants reported consulting their primary health care practitioner *M*(*SD*) = 4.44 (4.76) times in the past 12 months. Generally, the participants rated the quality of the relationship with their primary health care practitioner close to “totally appropriate” (*M* = 4.12 ± .69). The degree of insecure attachment manifested towards the lower extremity of the scale. The total effect of the mediation analyses was significant. Regardless, the indirect effect indicated a trend result with minimal effect sizes.

**Conclusion:**

The findings of the current study bridged the gap between attachment styles and healthcare utilization. Nonetheless, our results suggested insufficient support for the mediating role of the primary care physician in the relationship between attachment style and healthcare utilization. Considering the characteristics of the sample, this outcome may not apply in a clinical context. However, further research is needed to shed light in the revealed trends and indicate implications.

## Introduction

The attachment theory [1] provides a psychosocial framework for understanding the relationship between attachment-styles and health-related behaviors [2–4]. Since attachment processes are closely related to emotion regulation and coping behavior (e.g., illness [1, 5, 6];, they predict health behaviors [7] and outcomes [8, 9]. Attachment is a fundamental need [1] and is also perceived a stable trait [10] notably activated during vulnerable times. “Attachment styles” are conceptualized as interpersonal dynamics [1, 11–13] categorized as *secure* and *insecure* attachment-styles (i. e., dismissing, preoccupied, and fearful [1, 14]).

The present research is based on the concept of attachment-related anxiety and avoidance [13, 14]. These dimensions conceptualize self-regulation mechanisms for seeking emotional proximity to an attachment figure during stressful events (e. g., sickness and distress). Anxious attachment-style is related to “clingy” and “controlling” behaviors, while avoidant-attachment is linked to mistrust and reclusive of social relationships [15]. Consistent with this framework, past evidence demonstrates that insecure attachment styles are predictive of unhealthy behaviors [16–22]. However, both with different patterns health care utilization. On the one hand, anxious-attached patients display attention-seeking behavior and overuse health services [3, 23, 24]. On the other hand, avoidant-attached patients tend to underuse health care [15, 22, 25] and engage in self-treatment [24]. This pattern was also observed in the context of primary care. Ciechanowski et al. [26] found that (female) patients with preoccupied attachment had the highest primary care costs and utilization, whereas patients with fearful attachment the lowest.

A strong physician-patient relationship is paramount for effective treatment [27–30]. However, there are not many studies on how the quality of the dyadic physician–patient relationship impacts the use of primary health care practitioners (PCP) [31] and current results are mixed. Nonetheless, empirical evidences suggests that a better primary practitioner-patient relationship was positively correlated associated with increased consultations [31–34]. Fenton et al. [35] showed a similar outcome, reporting that a satisfactory physician-patient relationship was associated to higher overall healthcare utilization. On the other hand, Dinkel et al. [31] found that a strong family physician–patient relationship was not correlated with frequent visits to the PCP. Even so, these studies did not assess attachment, which is likely to affect the patient-physician-relationship [3, 36]. Since patients with insecure attachment styles show difficulties in the patient-physician relationship [37, 38] and mistrust health care providers [26, 39, 40], they might avoid visits to the doctor [40, 41].

Based on the presented background, one may assume that physicians, as the experts might be perceived as an attachment fig [37, 42]. As such, the PCP may active attachment pathways in the patient manifesting in a certain healthcare-seeking or avoidant behavior. Consequently, we hypothesized that the patient-physician relationship may mediate the link between the attachment-style and healthcare utilization patterns.

**Fig. 1:**
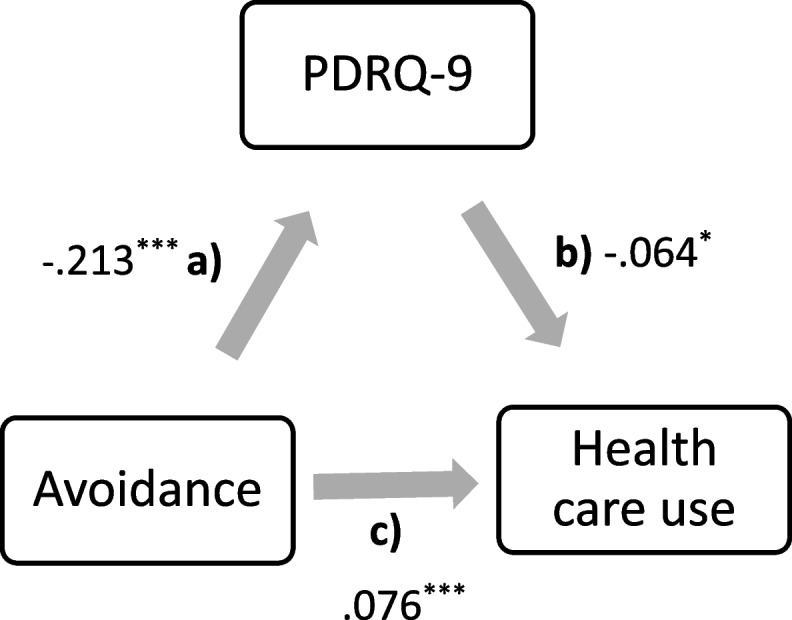
Mediation analysis: PDRQ-9 mediates the correlation between the attachment-style: avoidance and health care use (visits to the primary care practitioner in the last 12 months). Note: Indirect effect: (**a**) x (**b**). Direct effect: (**c**). Total effect: direct + indirect; *p* = ^***^ < .001; ^**^ = .005; ^=*^ < .05

**Fig. 2:**
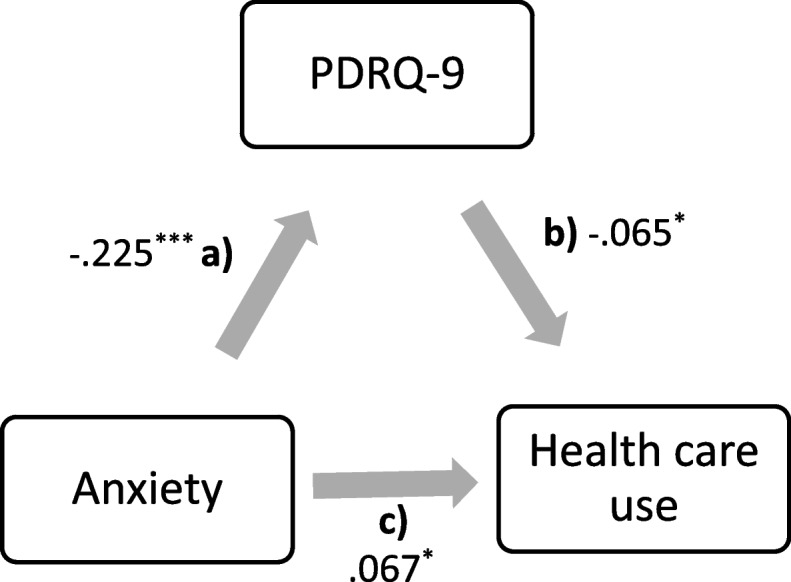
Mediation analysis: PDRQ-9 mediates the correlation between the attachment-style: anxiety and health care use (visits to the primary care practitioner in the last 12 months). Note: Indirect effect: (**a**) x (**b**). Direct effect: (**c**). Total effect: direct + indirect; *p* = ^***^ < .001; ^**^ = .005; ^=*^ < .05

In summary, research indicates that an individual’s attachment style impacts both the quality of their patient-physician relationship and healthcare utilization patterns. However, most studies focus on the individual dimension, rather than the dyadic level, i. e., exploring how insecure attachment relates to health behavior within patient-physician relationships. This gap leaves the role of the patient-doctor relationship in this process unclear. Hitherto, two studies evinced that the quality of the patient-provider relationship serves a mediator, however, between attachment and self-management in clinical samples [24, 43]. To the best of our knowledge this is the first study to examine this association in the context of PCP and in a representative sample of the German population. Based on the established correlation between attachment and health care use, we predicted that this correlation is mediated by the quality of the specific relationship with the PCP. To this end, we conducted mediational analysis (see Figs. 1 and 2).

## Methods

### Study participants

A representative sample of the German population was carefully selected with the assistance of a demographic consulting company (USUMA, Berlin, Germany). A total of *N* = 4360 participants were contacted to participate in the self-report survey. In total, *N* = 1852 participants did not collaborate with this self-report survey for several reasons (*n* = 647 unsuccessful attempts to contact, *n* = 591 declined to participate, *n* = 37 holiday break, *n* = 19 severe illness, *n* = 540 refused to finish the whole interview). The survey asked the participants whether they had a primary care physician (PCP). In the case of a positive response to this question, the person was asked to complete the Patient-Doctor-Relationship-Questionnaire-9 (PDRQ-9).

In total, *N* = 2508 individuals participated in the study (participation rate 58%) during June – July, 2013. Participants who did not visited their PCP were not included in the analyses. Participants with missing data in at least one of the items (*n* = 233) were excluded from the analysis. The final sample consisted of *N* = 2275 participants. The majority of the participants in the sample held German citizenship (96.7%) and were in average of 51 years (± 18). Further details about the sample can be found in Table 1, which provides a description of the participants’ characteristics.

**Table 1:** Demographic characteristics of the study population

Variable	Men *n* = 1031 (46.3%)	Women *n* = 1244 (54.7%)	Total *N* = 2275	Stat.
**Mean Age**	50.58 ± 18.07	50.87 ± 18.35	50.74 ± 18.22	no stat. Diff.
**Living in partnership**				X^2^ = 17.24 *p* < .0001 V = .087
yes	598 (58%)	613 (49.3%)	1.211 (53.2%)	
no	433 (42%)	631 (50.7%)	1.064 (46.8%)	
**Employment status**				X^2^ = 235.8 *p* < .0001 V = .322
Working	530 (51.4%)	345 (27.7%)	875 (38.5%)	
Working < 15 h per week	33 (3.2%)	249 (20%)	282 (12.4%)	
Training / house wife/man	81 (7.9%)	182 (14.6%)	263 (11.6%)	
unemployed	65 (6.3%)	60 (4.8%)	125 (5.5%)	
Retired	322 (31.2%)	408 (32.8%)	730 (32.1%)	
**Education**				X^2^ = 6.57 *p* < .04 V = .054
≤ 8 years	437 (42.6%)	520 (41.9%)	957 (42.2%)	
9–10 years	396 (38.6%)	531 (42.8%)	927 (40.9%)	
< 10 years	194 (18.9%)	191 (15.4%)	385 (17%)	
**Persons in household**	2.09 ± 1.05	2.06 ± 1.13	2.08 ± 1.09	no stat. Diff.
How many times have you been **unemployed**, including today				no stat. Diff.
0	583 (56.5%)	752 (60.5%)	1.335 (58.7%)	
1–2	316 (30.6%)	356 (28.6%)	672 (29.5%)	
3–30	132 (12.8%)	136 (10.9%)	268 (11.8%)	
**Household income in €**				X^2^ = 34.79 *p* < .0001 V = .124
< 1500	198 (19.2%)	338 (27.2%)	536 (23.6%)	
1500 < 2500	470 (45.6%)	581 (46.7%)	1.051 (46.2%)	
2500 < 3499	205 (19.9%)	208 (16.7%)	413 (18.2%)	
≥ 3500	158 (15.3%)	117 (9.4%)	275 (12.1%)	
**Part of the country**				no stat. Diff.
East Germany	197 (19.1%)	260 (20.9%)	457 (20.1%)	
West Germany	834 (80.9%)	984 (79.1%)	1.818 (79.9%)	
**Nationality**				no stat. Diff.
German	991 (96.1%)	1.208 (97.1%)	2.199 (96.7%)	
Other	40 (3.9%)	40 (3.9%)	76 (3.3%)	

### Procedures

The study participants were selected based on a random sample selection consisting in a multistage sampling. First, 258 sample point regions, covering rural and urban areas from all regions in Germany, were randomly drawn from the most recent political election register. The second stage was a random selection of household using the random route procedure (based on a starting address). The third stage was a random selection of household respondents using the Kish selection grid. The aim of the sampling procedure was to obtain a sample that was representative of the German population in terms of age, gender, and education. Participant selection based on electoral districts, a random-route procedure, and the Kish selection grid led to a sample representative of the German general population in terms of sex and age. Only participants with sufficient command of the German language were included in the study. Each respondent was visited by a trained interviewer who – after the respondent gave informed consent – collected information. All participants were informed of the study procedures, data collection, and anonymization of all personal data. Additionally, a detailed data privacy statement was delivered by the interviewer. The present study posed a low risk to the participants, as procedures such as medical treatments, invasive diagnostics or procedures causing psychological or social harm were not included in the present study. Therefore, according to German law, all participants provided verbal informed consent. Furthermore, the study was conducted in accordance to the guidelines of the ICMJE Recommendations for the Protection of Research Participants and the Helsinki Declaration as revised 2008. The study and procedure were approved by the institutional ethics review board of the University of Leipzig (Ethics Nr. 050/13–11,032,013). Furthermore, the study was executed according to the guidelines of the ICC/ESOMAR International Code of Marketing and Social Research Practice.

## Measures

The *Experiences in Close Relationships-Revised questionnaire* (ECR-RD8 [44];) measures attachment-related anxiety and avoidance with 8 items, e.g.: “I often worry that my partner will not want to stay with me” (*anxiety*); “I am comfortable sharing my private thoughts and feelings with my partner” (*avoidance*). The individual anxiety and avoidance scores are obtained by calculating the mean of the respective items. All items of the avoidance subscale are inverse coded. Items scores range from 1 “strongly disagree” to 7 “strongly agree”. Regarding the convergent validity of the subscale anxiety is moderately correlated with lower scores on the secure scale and higher scores on the preoccupied and fearful subscales of the Relationship Questionnaire (RQ [14];). Similarly, attachment avoidance correlated moderately with the RQ-subscales *secure* (negative correlation) and *fearful*. The correlation between attachment *avoidance* and RQ-*dismissing* was small. The reliability of the ECR-RD8 can be rated as high (ω = 0.87; anxiety and ω = 0.91; avoidance subscale).

The *Patient-Doctor Relationship Questionnaire* (PDRQ-9 [45, 46];) was originally developed as an assessment tool of the relationship between the PCP and the patient’s perspective [45]. The scale was adapted from an existing instrument based on the Helping Alliance Questionnaire (HAQ [47];), which is often applied in primary care and public health research. The PDRQ-9 is a unidimensional tool that evaluates the patient’s experience. The latter relate to several aspects of their relationship (e.g., time available, understanding, openness) using nine questions on a five-point Likert scale, ranging from “1 = not at all appropriate” to “5 = totally appropriate.” In a validation study [46], the patient-doctor relationship with a focus on the empathic style and availability of the doctor was assessed. A higher average score suggests a stronger relationship [45]. Past evidence shows good psychometric properties (e. g., α = .95 [45, 46, 48–50];.

### Healthcare utilization

To operationalize healthcare utilization, the participants were asked to respond if and how frequently they consulted their primary care practitioner in the last 12 months. Only participants who visited their PCP were included and the total number of visits in the past 12 months was then calculated. All items were assessed according to the National Health Interview and Examination Survey [51, 52]. This scoring system provided a quantifiable measure of the participants’ utilization of healthcare services and has been used in similar past studies [26, 31].

## Statistical analyses

The statistical analyses were performed with the Statistical Package for the Social Sciences (SPSS version 24.0) and *R* [53]. In the present study, we reported the mean and standard deviation of the examined variables (Table 2) and the Person moment correlation coefficients between the examined variables. To test our main hypothesis, we conducted a mediation analysis with attachment-style (i.e., anxious and avoidant respectively) as a predictor, the PDRQ-9 as the mediator and healthcare utilization as the outcome variable. Specifically, we built this mediation model in a structural equation model (SEM) using *lavaan* [54] to estimate (using the robust maximum likelihood estimator) and test path coefficients and the indirect effect. The *lavaan* package is a common tool for conducting structural equation modeling [54]. In this regard, it has been applied for mediation analysis [55] among other analyses. Healthcare utilization was operationalized by frequencies (number of consults in the past 12 months), as reported by the participants (see Table 2.).

**Table 2:** Means and Standard Deviations for the analyzed dimensions

	*M*	*SD*	*Median*	*Min*	*Max*
Anxiety	2.40	1.28	2.16	1	7
Avoidance	2.89	1.68	2.5	1	7
PDRQ-9	4.12	.69	4.11	1	5
Health care use	4.44	4.76	3	1	52

Anxiety and Avoidance represent insecure attachment-styles that were measured by the subscales of the ECR-RD8

## Results

The characteristics of the sample are displayed in detail in Table 1. *Healthcare utilization*: in average the participants reported consulting their PCP *M*(*SD*) = 4.44 (4.76) times. The reported visits ranged from 1 to 52. In average the patients rated the *quality of the relationship* with their PCP close to “totally appropriate” (*M* = 4.12 ± .69). Concerning avoidant and anxious attachment, both were rather on the lower end of the scale (see Table 2). The total effect of the *mediation analyses* was significant. However, the indirect effect was not and indicates only a trend result (see Table 3 and Figs. 1, 2). In general, the effect sizes of the mediation analyses were minimal. This outcome suggested that the patient-physician relationship (PDRQ-9) may not mediate the correlation between attachment-style and health care utilization (Avoidance: *β* = .014, *p* = .054 and Anxiety: *β* = .015, *p* = .051).

**Table 3:** Mediation analysis

	*β*	*Estimate*	*Standard error*	*p*	*R*^*2*^	*d*
*Regressions*						
**Avoidance**						
Avoidance → PDRQ-9	−.213	−.089	.009	<.001	.045	.436
PRDQ-9 → Utilization	−.064	−.427	.215	.047	.004	.128
Avoidance → Utilization	.076	.212	.060	<.001	.006	.152
Indirect effect	.014	.038	.020	.054	.001	.028
Total effect	.090	.250	.059	<.001	.008	.181
**Anxiety**						
Anxiety → PDRQ-9	−.225	−.122	.012	<.001	.051	.462
PRDQ-9 → Utilization	−.065	−.436	.216	.044	.004	.130
Anxiety → Utilization	.067	.243	.097	.012	.004	.134
Indirect effect	.015	.053	.027	.051	.000	.030
Total effect	.082	.297	.094	.002	.007	.165

*β =* is the standardized beta-weight. Arrows (→) are indicative of predictions

## Discussion

The purpose of the study was to assess whether the patient-physician-relationship mediates the association between attachment-style and healthcare utilization in the context of primary care. In sum, our results demonstrated a significant and positive correlation between insecure attachment (i. e., avoidance, anxiety) and health care use. Furthermore, our data suggested that insecure attachment is related to a negative experience of the patient-physician-relationship. Nevertheless, in the present examination our hypothesis was not supported by our data: The minimal effect sizes implied that the patient-physician-relationship may not have a substantial impact on the link between attachment-style and health care use in the context of primary care. The revealed positive correlation between insecure attachment and health care utilization is not in line with past studies. The latter reporting that anxious-attached patients tend to over use health care, while avoidant-attached had the lowest health care utilization in primary care [26]. Even if the results of Ciechanowski et al. [26] align with outcomes in the context of general health care utilization [23], it is important to emphasize their relevance to a specific female sample. Weber et al. [56] found that females showed higher attachment anxiety, while males higher attachment avoidance. Furthermore, our findings pertaining the negative experienced patient-physician-relationship among insecure-attached individuals confirmed past findings describing similar negative interpersonal dynamics [37, 38, 40, 41]. Lastly, the result of our mediation analysis stands in contrast to previous research evidencing a significant mediating role of the patient-physician-relationship, regardless, between attachment and self-management [24, 43]. While self-management may reflect health care use, it encompasses a range of behaviors besides visiting the PCP, as examined in our study. Beyond that, the studied population by Brenk-Franz et al., [24] was older (50–85 yrs.) and had diabetes, which only affects approx. 7,2% of the German population [57], clearly differing from our representative sample. With regards to the mediation analysis, the limited effect of the patient-physician relationship on the attachment-style and healthcare utilization in primary care can be presumably explained by the characteristics of our sample. As opposed to past comparable studies, our participants scored low on both scales of insecure attachment and rated their relationship to their PCP as satisfactory. Since most of the comparable studies examined non-representative samples, it is possible that the mediating effect of the patient-physician relationship on attachment and health care use unfolds in the context of greater score-values in the examined variables (e. g., higher scores in insecure attachment and health care use). Indeed, patterns of emotional regulation and consequently health-related behavior can be perceived as the result of a hyper-activated attachment system, which is activated during vulnerable or threatening times [1, 13, 58]. Since 3–4 visits per year to the PCP (as reported by our participants) are below the threshold of frequent attendance [59], one may assume less perceive threat by our participants. Equally, factors such as physician-related variables (e.g., attachment-style, sympathy) might also affect patient’s behavior [60, 61] and thus, visits to their PCP. However, these factors were not part of the present study. Future studies, might benefit in including these variables that might have also impacted the quality of the relationship between the patient and their PCP and thus health care use. Taken together, our findings are preliminary and should be interpreted cautiously. One of the strengths of our study lies in the considerable size and representativeness of the study sample. On the other hand, the minimal effect sizes constrain the interpretation of our results. Even so, our findings are a useful reference for future studies. A further limiting factor pertains the data referent to the attachment-styles of individuals, who were not in a current relationship (or dating). Consequently, affecting how this status reflects on health care use. Further research might benefit from evaluating the attachment-style of the physician and also from investigating how the current relationship status of the patient might impact health care use. By elucidating the role of the patient-physician relationship, strategies that consider patients’ attachment styles can be tailored to foster more positive and supportive patient-physician relationships, leading to fruitful patient experiences and improved health outcomes [62].

In conclusion, our results suggested limited evidence regarding the mediating role of the PCP between attachment-style and health-care utilization. However, this research question remains highly relevant. In Germany, the PCP as the primary health provider plays a key role in managing further interventions for the patients and delivering effective treatment. Hence, improving the working relationship might optimize both, patient’s health and doctors’ resources by reducing burden on the healthcare system in the long run. Therefore, further studies are warranted to shed light in the revealed trends and be able to establish the implications of the observed outcomes.

## Data Availability

The datasets used and/or analyzed during the current study are available from Prof. Katja Petrowski: kpetrows@uni-mainz.de on reasonable request.
